# In a model of SAH-induced neurogenic fever, BAT thermogenesis is mediated by erythrocytes and blocked by agonism of adenosine A1 receptors

**DOI:** 10.1038/s41598-021-82407-w

**Published:** 2021-02-02

**Authors:** Domenico Tupone, Justin S. Cetas

**Affiliations:** 1 Department of Biomedical and Neuromotor Science, University of Bologna, 40126 Bologna, Italy; 2 Department of Neurological Surgery, Oregon Health and Science University, 3181 SW Sam Jackson Park Road, Portland, OR 97239-3098 USA; 3 Portland VA Health Care System, Portland, OR USA

**Keywords:** Homeostasis, Aneurysm

## Abstract

Neurogenic fever (NF) after subarachnoid hemorrhage (SAH) is a major cause of morbidity that is associated with poor outcomes and prolonged stay in the neurointensive care unit (NICU). Though SAH is a much more common cause of fever than sepsis in the NICU, it is often a diagnosis of exclusion, requiring significant effort to rule out an infectious source. NF does not respond to standard anti-pyretic medications such as COX inhibitors, and lack of good medical therapy has led to the introduction of external cooling systems that have their own associated problems. In a rodent model of SAH, we measured the effects of injecting whole blood, blood plasma, or erythrocytes on the sympathetic nerve activity to brown adipose tissue and on febrile thermogenesis. We demonstrate that following SAH the acute activation of brown adipose tissue leading to NF, is not dependent on PGE_2_, that subarachnoid space injection of whole blood or erythrocytes, but not plasma alone, is sufficient to trigger brown adipose tissue thermogenesis, and that activation of adenosine A1 receptors in the CNS can block the brown adipose tissue thermogenic component contributing to NF after SAH. These findings point to a distinct thermogenic mechanism for generating NF, compared to those due to infectious causes, and will hopefully lead to new therapies.

## Introduction

Subarachnoid hemorrhage (SAH) is a devastating form of stroke, with release of blood into the subarachnoid (SA) space, most commonly from aneurysm rupture or traumatic brain injury. Aneurysmal SAH accounts for 3% of all strokes in the US^1^, predominately affects a younger population, and is associated with higher morbidity and mortality. A broad constellation of diverse homeostatic systems are impacted (both acutely and delayed) by SAH, including dysfunction in thermoregulatory, respiratory, cardiovascular, and cerebral blood flow regulation. This autonomic imbalance complicates treatment, exacerbates secondary injuries and requires prolonged hospitalization with increased medical costs^2^. Fever in SAH patients is quite common (about 40% of SAH patients) and is associated with poor outcomes and increased mortality^3,4^. Prevention of secondary injury improves outcomes after SAH, however, there are no effective treatments for neurogenic fever (NF). Further, it is estimated that only 3.6% of patients have septic fever (SF). In contrast to patients with septic fever, febrile SAH patients rarely respond to current antipyretic treatments, such as COX inhibitors^5^. In addition, the lack of an effective medical therapy has led to the development and clinical use of external and internal cooling devices^5,6^ which inadvertently activate thermogenic responses such as shivering, that further increase the metabolic demand for oxygen and the need for sedation. Sedation and increased oxygen demand can worsen secondary injury through an increased risk of aspiration pneumonia, hypoxia, and increased oxygen consumption as well as an increase in the sympathetic surge already elevated in SAH patients^7,8^. Clearly, an improved understanding of the trigger mechanisms in NF, the thermoeffector tissues generating NF, and of the central neuronal circuitry responsible for the NF in SAH could lead to a better therapeutic approach to controlling body temperature after SAH and have a significant impact on survival and recovery in SAH patients.

Few studies have addressed the mechanisms for the generation of NF^9,10^, but rather there are case reports or clinical trials testing hypothermic devices^11–18^ or simple descriptive studies^3,4,19–21^ relating poor outcomes to the presence of NF^22–26^. Indeed, there are no data on the central neuronal mechanism(s) underlying the generation of NF, or on the molecular trigger for NF in SAH patients. In contrast, the neuronal circuit involved in the generation of septic fever in response to inflammation or infection has been well described in rodents^27^ and the same neuronal circuit is likely present in human^28^. The activation of this circuit and the resulting elevation of core body temperature is due to the action of inflammatory mediators, most prominently prostaglandin E_2_ (PGE_2_), on this neuronal thermoregulatory circuit. Specifically, the immune cell activation of the enzyme, cyclooxygenase (COX), increases production of PGE_2_, which acts on EP_3_ receptors in the preoptic area (POA) of the hypothalamus, leading to increased activity of thermoregulatory circuit neurons in the dorsal medial hypothalamus (DMH) and the rostral raphe pallidus (rRPa) that promote fever by driving brown adipose tissue (BAT) and shivering thermogenesis as well as skin vasoconstriction (reviewed in Ref.^27^).

Here we employ a rodent model of experimental SAH to show that injection of whole blood into the subarachnoid (SA) space of the basal cisterns is sufficient to cause an acute NF, and that it is the erythrocytes and not the plasma component that is responsible for this fever. Further, we show that BAT is activated after experimental SAH and that PGE_2_ does not mediate this fever response. Further, we suggest an alternative and novel approach to block this indomethacin-resistant fever in the rat.

## Results

### Experimental SAH induces NF

**Figure 1:**
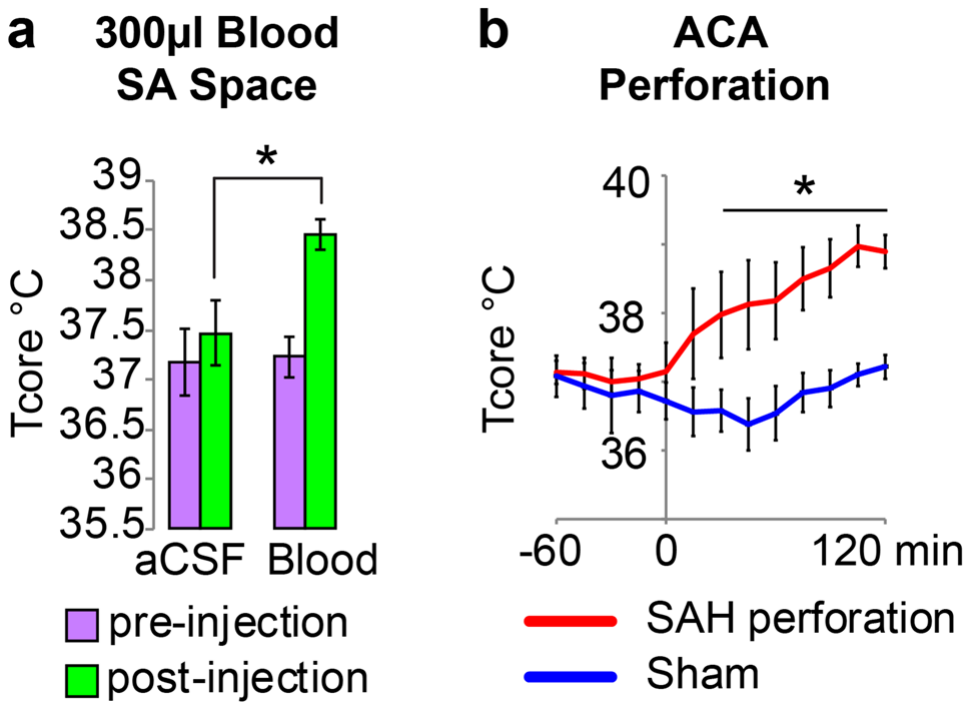
NF is triggered by release of blood into SA space. (**a**) Group data representing the changes body temperature (T_CORE_) between baseline (pre-injection) and 120 min following (post-injection) direct injection of blood or artificial cerebrospinal fluid (aCSF), into the subarachnoid (SA) space. The injection of blood, but not aCSF, elicited an increase in T_CORE_. (**b**) Group data representing the time course of the changes in T_CORE_ following perforation of the anterior cerebral artery (ACA), or the sham perforation procedure. The extravascular release of blood into the SA space, produced by the perforation of the ACA, led to a significant increase in T_CORE_. *p < *0.05.*

We employed two techniques in free behaving rats to produce experimental SAH and NF (Fig. 1a,b): (a) direct injection of blood into the SA space at the pre-chiasmatic cistern level and (b) perforation of the internal cerebral artery (ICA) bifurcation at the circle of Willis.

Direct injection of autologous blood (300 µl), but not vehicle (300 µl of aCFS + heparin), into the SA space at the pre-chiasmatic cistern level lead to an increase in T_CORE_ (ΔT_CORE_ = 1.36 ± 0.1 °C, from a baseline of 37.1 ± 0.2 °C, t = 2.756, n = 14, p < 0.05 Bonferroni post-hoc test, Fig. 1a). The T_CORE_ increase, reached its maximum (38.5 ± 0.2 °C) 120 min following the injection of blood into the SA space. T_CORE_ values at baseline were not different between treatment groups (t = 0.1198, p > 0.05 Bonferroni post-hoc test).

ICA perforation also induced a NF. T_CORE_ increased rapidly following ICA perforation, and became significantly different from that of the sham control at 30 min after the treatment (Fig. 1b, T_CORE_: 38.13 ± 0.6, t = 3.312, p < 0.05 n = 10, Bonferroni post-hoc test) and reached its maximum (T_CORE_: 38.9 ± 0.2 °C) in 2 h. T_CORE_ values at baseline were not different among treated groups (t = 0.3701, p > 0.05 Bonferroni post-hoc test).

### Brown adipose thermogenesis contributes to the NF following experimental SAH, but the NF is not mediated by PGE_2_

**Figure 2:**
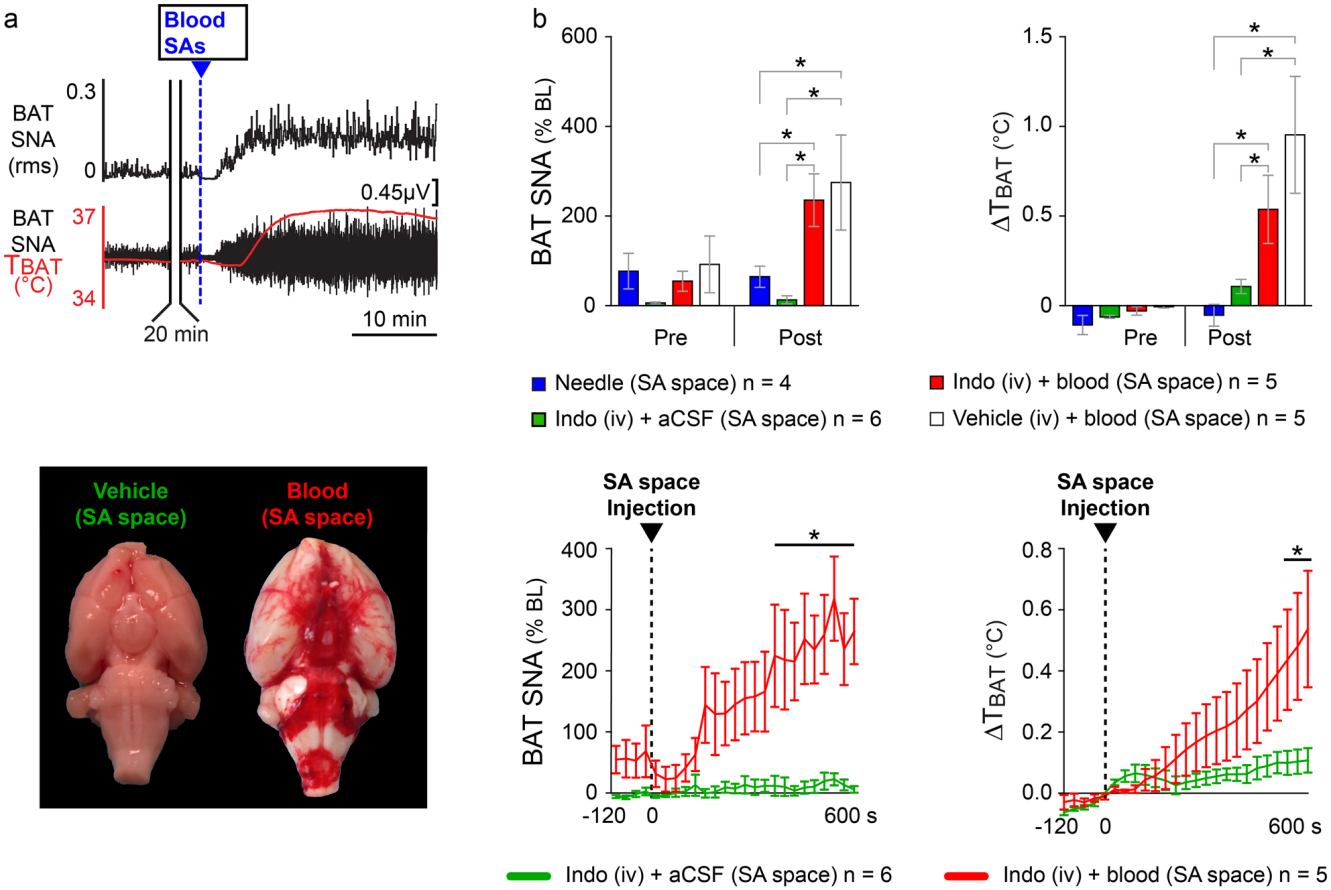
Pretreatment with indomethacin does not prevent the increases in BAT SNA and BAT thermogenesis induced by direct injection of blood into the SA space. (**a**) Top panel: responses in BAT SNA and T_BAT_ elicited by the injection of blood in the SA space of a rat pretreated with indomethacin. The injection of blood produced a prompt increase in BAT SNA and BAT thermogenesis (increase in T_BAT_). Lower panel: extravascular blood following the injection of blood into the SA space (right brain) compared with the ventral surface of the brain of a rat injected with vehicle into the SA space (left brain). (**b**) Top panels: group data showing the effects on BAT SNA and BAT thermogenesis (T_BAT_) resulting from insertion of the injection needle into the SA space (blue bars); injection of aCSF into the SA space after indomethacin (iv) pretreatment (green bars); injection of blood into the SA space after indomethacin (iv) pretreatment (red bars); or injection of blood in rats pretreated with saline vehicle (iv) (white bars). Bottom panels: time courses of the effects on BAT SNA and T_BAT_ following the injection of either blood or aCSF into the SA space. *p < 0.05.

We determined the contribution of increased BAT SNA and BAT thermogenesis as well as the contribution of the endogenous pyrogen, PGE_2_, in the induction of NF. Pretreatment with indomethacin (2 mg/kg, i.v.) blocks the production of the endogenous pyrogen, PGE_2_, allowing an assessment of its role in mediating the NF following SAH. In anesthetized rats pretreated with indomethacin, to block PGE_2_ synthesis, injection of 300 µl of blood into the SA space induced an immediate (within 3.1 ± 1.1 min) increase in BAT SNA that was, at 10 min following the treatment, 333% greater than BAT SNA level measured before the injection of blood (BAT SNA pre-blood injection: 55.2 ± 22.2% BL; post-blood injection BAT SNA: 239.3 ± 56.2% BL; n = 5, t = 4.177, p = 0.0140 t-test). The increase in BAT SNA produced an increase in T_BAT_ (ΔT_BAT_: + 0.6 ± 0.2 °C, from a baseline of 34.6 ± 0.4 °C, n = 5, p = 0.0275 t-test, Fig. 2b), reflecting the activation of BAT thermogenesis.

The peak of the increased BAT SNA triggered by blood into the SA space was significantly greater than the peak level of BAT SNA measured at 10 min following either needle insertion (peak BAT SNA_(blood)_: 239.3 ± 56.2% BL vs peak BAT SNA_(needle)_: 61.8 ± 25.4% BL, t = 2.353, p < 0.05 n = 4, Bonferroni posthoc test, Fig. 2b) or vehicle injection into the SA space (peak BAT SNA_(blood)_: 239.3 ± 56.2% BL vs peak BAT SNA_(vehicle)_: 13.2 ± 8.9% BL, t = 3.320, p < 0.01, n = 6, Bonferroni posthoc test, Fig. 2b). This finding was paralleled by the effects of injecting whole blood into the SA space on BAT thermogenesis which was significantly greater than ΔT_BAT_ measured 10 min after needle insertion T (ΔT_BAT(blood)_: + 0.6 ± 0.2 °C vs ΔT_BAT(needle)_: − 0.05 ± 0.06 °C, t = 3.268, p < 0.01, n = 4, Bonferroni posthoc test, Fig. 2b) or vehicle injection into the SA space (ΔT_BAT(blood)_: + 0.6 ± 0.2 °C vs ΔT_BAT(vehicle)_: + 0.1 ± 0.04 °C, t = 2.658, p < 0.05, n = 6, Bonferroni posthoc test, Fig. 2b).

The peak level of BAT SNA and the ΔT_BAT_ elicited by injection of 300 µl of blood into the SA space were not different between rats pretreated with vehicle and those pretreated with indomethacin (vehicle (iv) + blood (SA space) peak BAT SNA: 275.2 ± 106.0% BL vs indomethacin (iv) + blood (SA space) peak BAT SNA: 239.3 ± 56.2% BL, t = 0.5043, p > 0.05 n = 9, Bonferroni posthoc test, Fig. 2b; vehicle (iv) + blood (SA space) ΔT_BAT_ =  + 0.9 ± 0.3 °C vs indomethacin (iv) + blood (SA space) ΔT_BAT_ =  + 0.6 ± 0.2 °C, t = 2.300, p > 0.05, Bonferroni posthoc test, Fig. 2b). These results indicate that iv indomethacin did not affect either the amplitude of the BAT activation or the increase in BAT thermogenesis during the NF accompanying experimental SAH.

### RBC rather than plasma produces NF

**Figure 3:**
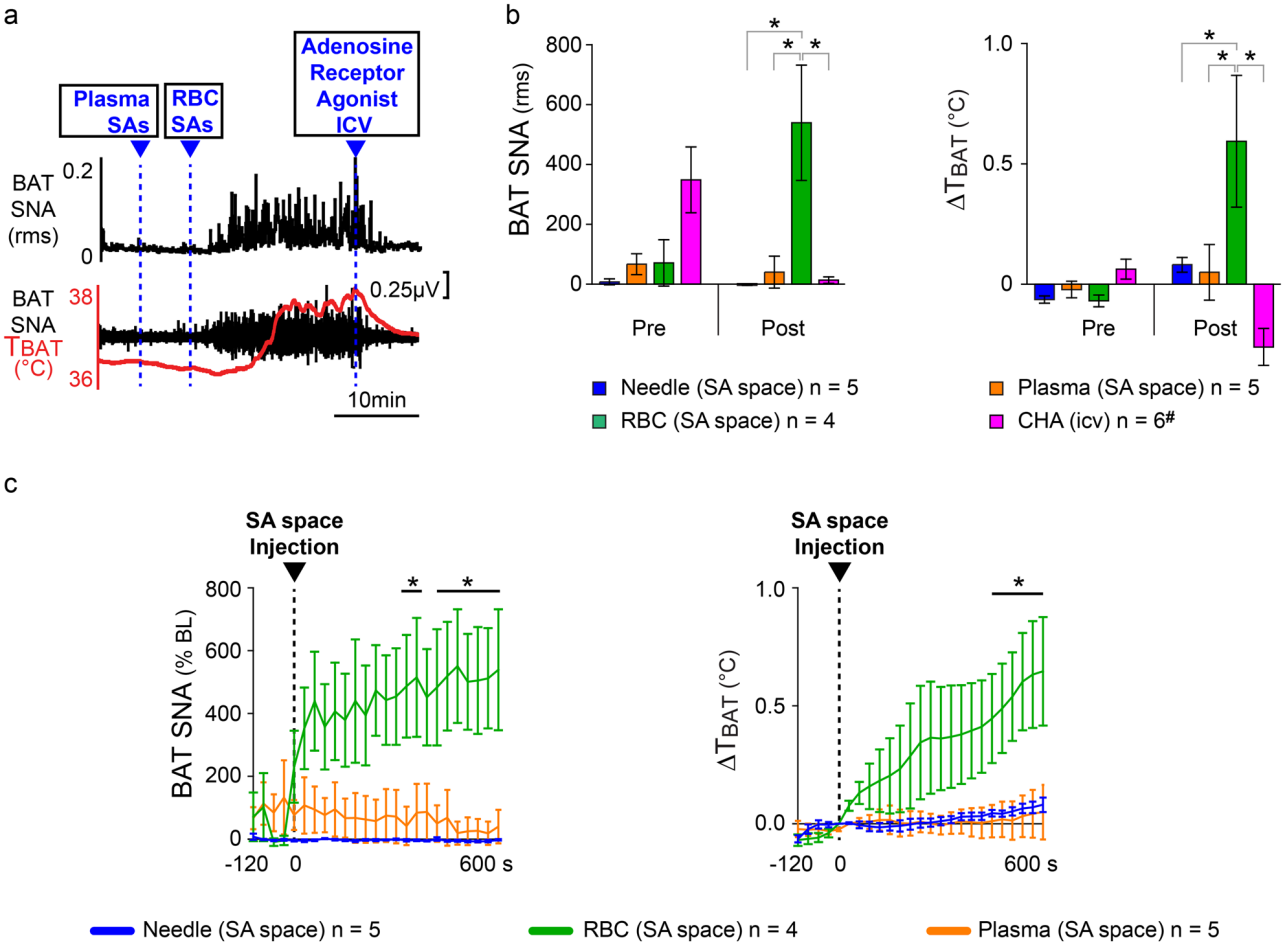
Injection of the red blood cell (RBC) fraction, but not plasma, into the SA space activates BAT SNA and BAT thermogenesis, contributing to NF. (**a**) An example of the effects on BAT SNA and T_BAT_ of injection of plasma or of red blood cells in the SA space (SAs). The injection of red blood cells, but not plasma, into the SAs elicited a prompt increase in BAT SNA and BAT thermogenesis (increase in T_BAT_). ^#^The increases in BAT SNA and BAT thermogenesis elicited by either injection of red blood cells (n = 3) or blood (n = 3) in the SAs were blocked by the intracerebroventricular (icv) injection of the A1 adenosine receptor agonist, CHA (n_tot_ = 6). (**b**) Group data showing the effect on BAT SNA and BAT thermogenesis resulting from insertion of the needle into the SA space (blue bars); injection of plasma into the SA space (orange bars); injection of RBC into the SA space (green bars); or injection of CHA icv (pink bars). (**c**) Time course of the effects on BAT SNA and T_BAT_ following the insertion of the needle, or the injection of plasma or of RBC into the SA space. *p < 0.05.

To determine whether the RBC or plasma component of whole blood contains the necessary molecular trigger for the NF following experimental SAH, we separated autologous blood samples into the plasma and RBC fractions and injected these separately into the SA space to determine their contribution to the increases in BAT SNA and BAT thermogenesis elicited by experimental SAH. Injection of re-suspended erythrocytes into the SA space was done approximately 10 min following injection of plasma (Fig. 3).

At 10 min following injection of autologous blood plasma into the SA space, BAT SNA and T_BAT_ were not different from the pre-injection control values (BAT SNA: 94.6 ± 57.3% BL prior to plasma injection vs 40.4 ± 53.5% BL at 10 min after plasma injection, n = 5, p = 0.0598, t-test; T_BAT_: 35.0 ± 0.7 °C prior to plasma injection vs 35.0 ± 0.6 °C at 10 min after plasma injection, n = 5, p = 0.6360, t-test, Fig. 3b). In contrast to the injection of autologous blood plasma, injection of resuspended autologous RBC into the SA space induced a prompt increase of BAT SNA and BAT thermogenesis (Fig. 3a,c). At 10 min following injection of resuspended RBC into the SA space, BAT SNA increased by 359% from a control value of 71.2 ± 77.3% BL to a peak of 539.6 ± 192.1% BL, n = 4, p = 0.0249 t-test, Fig. 3), causing an increase in BAT thermogenesis (ΔT_BAT_: + 0.7 ± 0.2 °C, Fig. 3, n = 4, p = 0.0295, t-test). Note one animal was removed from the analysis due to the loss of nerve recording, then n = 4 in the group “injection of resuspended RBC into the SA space”. The peak levels of BAT SNA and the resulting increases in BAT thermogenesis occurring 10 min after the injection of autologous RBC were not different from those following injection of whole autologous blood (peak BAT SNA_(blood)_: 239.3 ± 56.2% BL n = 5 vs peak BAT SNA_(RBC)_: 539.6 ± 192.1% BL n = 4, p = 0.1398, t-test; ΔT_BAT(blood)_: + 0.4 ± 0.2 °C, n = 5 vs ΔT_BAT(RBC)_: + 0.6 ± 0.3 °C, n = 4, p = 0.5823, t-test).

The peak level of the increased BAT SNA triggered by autologous RBC was significantly greater than the peak level of BAT SNA measured at 10 min following either needle insertion (peak BAT SNA_(RBC)_: 539.6 ± 192.1% BL vs peak BAT SNA_(needle)_: − 1.772 ± 2.0% BL, t = 4.553, p < 0.001 n = 5 Bonferroni post-hoc test) or plasma injection (peak BAT SNA_RBC_: 539.6 ± 192.1% BL vs peak BAT SNA_(plasma)_: 40.41 ± 53.49% BL, t = 4.198, p < 0.001, n = 5, Bonferroni post-hoc test), into the SA space (Fig. 3b). The increases in BAT SNA triggered by autologous RBC resulted in increases in BAT thermogenesis which were significantly greater than the ΔT_BAT_ measured 10 min after needle insertion (ΔT_BAT (RBC)_: + 0.7 ± 0.2 °C vs ΔT_BAT(needle)_: + 0.08 ± 0.03 °C, t = 3.586, p < 0.01, n = 5, Bonferroni post-hoc test) or plasma injection (ΔT_BAT (RBC)_: + 0.7 ± 0.2 °C vs ΔT_BAT (plasma)_: + 0.05 ± 0.1 °C, t = 3.808, p < 0.01, n = 5 Bonferroni post-hoc test), into the SA space (Fig. 3b).

### Central activation of adenosine receptor reversed the increases in BAT SNA and BAT thermogenesis induced by experimental SAH

Activation of central adenosine A1 receptors (A1AR) by icv injection of CHA produces a potent inhibition of BAT thermogenesis^29–31^. Thus, we sought to determine if a similar ICV injection of CHA would block the increases in BAT SNA and BAT thermogenesis during the NF following experimental SAH.

Following the onset of NF elicited by injections of autologous whole blood or of autologous resuspended RBC into the SA space, ICV injection of CHA, reversed the evoked increases in BAT SNA and in BAT thermogenesis (Fig. 3a,b). ICV injection of CHA decreased BAT SNA from the post-SA space injection (blood: n = 3 and RBC: n = 3) level of 349 ± 109.9% BL to a nadir of 13.8 ± 10.64% BL (n = 6, p = 0.0063, t-test). The CHA-evoked decrease in BAT SNA was accompanied by a decrease in BAT thermogenesis (ΔT_BAT_: − 0.33 ± 0.09 °C, n = 6, p = 0.0020, t-test).

## Discussion

NF commonly occurs soon after (i.e., acute NF) SAH and can accompany delayed cerebral ischemia and angiographic vasospasm^32–34^. Numerous clinical studies have documented a strong correlation between the presence of fever and increased morbidity after SAH^33,35,36^. The underlying mechanisms of NF are unknown. We have combined measurements of BAT thermogenesis^29,37^ with two rodent models of SAH: (a) direct injection of autologous blood into the SA space at the pre-chiasmatic cistern level^38–40^ and (b) intracranial artery perforation^41–43^; to study the underlying mechanisms of the acute NF after SAH. In both cases, blood in the SA space induced an increase in BAT thermogenesis and in core body temperature that developed over two hours. This SAH-induced elevation in temperature, above the normal value of 37.5 °C (fever), was similar to the elevations in body temperature resulting from septic infection or administration of bacterial lipopolysaccharide (LPS)^44–46^.

We demonstrated, that the same stimulus (direct injection of autologous blood in the SA space) that elicited NF in freebehaving rats, was able to trigger BAT SNA and thermogenesis in anesthetized and curarized rats (no muscle thermogesis). This observation, supports the contribution of BAT thermogenesis to the increase in body temperature generated by experimental SAH. The role of BAT thermogenesis in mediating septic fever^8,27,47–52^ is well established in rodents^48–60^ and was reported in rabbit^61^, although a few recent studies in genetically-modified mice suggest that BAT thermogenesis may play a more limited role in the mouse febrile response to LPS^51,62,63^. However, its role in human febrile responses remains to be determined. Nonetheless, it is well established that BAT is present in adult humans and is strongly activated by cold exposure^64,65^, and the central nervous system regulation of BAT thermogenesis appears to share some of the same pathways as that described in rodents^27,28^, supporting a role for BAT activation in the human febrile response.

Febrile increases in body temperature result from increased heat retention due to cutaneous vasoconstriction and increased heat production from thermogenesis in BAT and from shivering in skeletal muscle^27,60^. However, the thermoeffector mechanisms contributing to the hyperthermia (i.e., NF) associated with SAH remain unknown. We provide here the first demonstration that activation of BAT thermogenesis contributes to at least the early phase of NF during experimental SAH. Although our experimental conditions (anesthesia and paralysis) did not allow simultaneous assessment of the potential roles of increased cutaneous vasoconstriction and shivering in the NF of experimental SAH, we expect that, as in septic fever, the recruitment of these additional thermoeffectors would have increased, and perhaps prolonged the NF during experimental SAH. Indeed, increased shivering is a frequent complication of clinical fever control (cooling blankets) in SAH patients^66^.

While PGE_2_ is a well-known mediator for septic fever^8^ its role in the induction of human NF has been controversial^67–69^. PGE_2_, the final driver of the fever proinflammatory mediator cascade^27,70–72^, acts in the POA to trigger the activation of BAT and shivering thermogenesis in septic fever in rodents^8,27,56,73,74^. Here, we have confirmed recent data^75^ that eliminates a role for PGE_2_ in the generation of NF in experimental SAH. We demonstrate that the activation of BAT SNA and BAT thermogenesis by experimental SAH is not prevented (Fig. 2) by indomethacin, a well-known COX 1–2 inhibitor that blocks PGE_2_ production^76^. The absence of a role for PGE_2_ in the activation of BAT thermogenesis in experimental SAH is also indicated by the rapid onset of the increase in BAT SNA after the injection of whole blood or RBC into the SA space (Figs. 2, 3). The synthesis of PGE_2_ requires several minutes, once triggered by LPS injection^27,59^, and this long synthesis time is not compatible with the rapid onset of BAT SNA recorded in our experiments. We can also exclude the possibility that our blood injection was contaminated with PGE_2_ (perhaps produced during surgical procedures), since injection of plasma (Fig. 3), which would have held any PGE_2_ in the original blood sample, did not produce an activation of BAT thermogenesis. Overall, our data indicate that PGE_2_ is not the primary mediator of NF induced by experimental SAH. These findings are consistent with clinical observations that ibuprofen and other NSAIDs are clinically ineffective in treating NF after SAH in patients^77–79^.

However, while our results show that PGE_2_ is not the main mediator of the early onset of NF, our study was not designed to detect a potential minor contribution of PGE_2_ to NF. Furthermore, our study only looked at the initial stage of NF in experimental SAH and therefore we cannot exclude the potential contribution that PGE_2_ could make to supporting later (days) phases of NF. It is important to highlight that hyperthermia in SAH patients could result from different mechanisms (e.g. infectious and non-infectious, early and late), acting at different stages of the SAH insult, and that these might require different types of treatment. This suggests that a more cautious diagnosis is required to determine what is causing the febrile response, and at which stage (early, late) of the disease the hyperthermia is occurring, to determine the best treatment.

To begin to identify the molecular trigger(s) for the NF evoked by experimental SAH, we compared the thermogenic effects of whole blood with those of its main components, washed RBCs and plasma. We found that RBCs, but not plasma, injected into the SA space, induced a robust rise in BAT SNA and BAT thermogenesis (Fig. 3). This finding suggests that the RBC membrane contains a molecule that directly or indirectly drives the activation of BAT thermogenesis, perhaps through an interaction with neurons^57,80^ in the hypothalamic fever center, during the onset of NF in experimental SAH.

We demonstrated that needle insertion into the SA space was without effect on BAT SNA or BAT thermogenesis, indicating that the BAT activation stimulated by experimental SAH was not the result of tissue disruption, or lesion of hypothalamic structures by mechanical means. Although hemoglobin can contribute to the generation of NF^9^, injection of hemoglobin into the SA space is unlikely to have contributed to our results. Not only did we use tinted plasma as an exclusion criterion for our plasma samples, but plasma injection did not produce an increase in BAT SNA or BAT thermogenesis. In addition, our control experiments with needle insertion alone, likely resulted in some disruption of local brain blood vessels and thus a potential leakage of a small amount of blood and hemoglobin into the SA space, but these were without effect on BAT SNA or BAT thermogenesis. Taken together, these results demonstrate that the RBC membrane, but not plasma, contains the molecular trigger responsible for the rapid increase in BAT thermogenesis that contributes to the early stage of NF in experimental SAH.

ICV injection of the A1AR agonist, CHA, completely blocked the activation of BAT thermogenesis in experimental SAH. Since CHA, able to induce a state of deep hypothermia^29,30^, is a potent inhibitor of central thermogenic pathways^29,30,81–83^, it may be that the BAT activation during the NF in experimental SAH arises through activation of the same canonical hypothalamic and medullary thermoregulatory circuits that underly the responses to cold exposure and to inflammation^74^. Although, the CHA inhibitory effect observed on BAT activation during the NF in experimental SAH, in not directly related to the triggering mechanisms for NF, its action somewhere along the pathway for thermogenesis^29,82^ remain of interest, as it suggest that the mechanism underlying the CHA effect, could be a useful intervention strategy to block NF. Recently, a Thermoregulatory Inversion mechanism, triggered by activation of A1AR agonist, has been described^31^ that could induce a hypothermic, torpor-like state^29^ that may provide effective management of drug-resistant fevers^27,82,84–86^. Such therapeutic approaches that act on central thermoregulatory pathways may prove useful in the treatment of NF after SAH.

In conclusion, we have demonstrated that a PGE_2_-independent activation of BAT thermogenesis contributes to the hyperthermia following experimental SAH, and that this activation is triggered by a molecule (yet to be uncovered) within the membrane of the RBC, rather than a mediator in the plasma fraction of whole blood. Our demonstration of the effectiveness of the central activation of A1AR in blocking the BAT activity component of the NF during experimental SAH suggests a new pharmacological approach with potential utility in the management of drug-resistant fevers.

## Materials and methods

### Animals

Male Wistar rats (300–400 g, Charles River Laboratories) were maintained in a standard 12 h/12 h, light/dark cycle with ad libitum access to standard chow and water. Experiments were performed in accordance with the *Guide for the Care and Use of Laboratory Animals,* Eighth Edition (National Research Council, National Academies Press, 2010) and protocols were approved by the Institutional Animal Care and Use Committee of Oregon Health & Science University.

### Surgical procedure and experimental protocol for injection model of experimental SAH

Seven days prior to the experiment, rats were anesthetized with 2% isoflurane in 100% O_2_ equipped with a temperature recording device (IPTT-300; Bio Medic Data Systems) that was implanted in the interscapular area in close apposition to the dorsal muscle layer and beneath BAT, in order to monitor temperature during the experimental procedures. Such temperature readings provide both an indication of rapid changes in BAT temperature (T_BAT_) as well as a long-term measurement of core temperature (T_CORE_). Animals were placed in a stereotaxic frame with the incisor bar positioned − 4 mm below interaural zero. A burr hole was drilled between the two frontal bones to gain access to the basal cisterns of the brain. An Eppendorf tube (100 µl) in which the bottom part was cut away, was secured over the burr hole to the skull surface, with dental acrylic. The cap of the Eppendorf was used to gain fast and easy access to the burrow hole, for the injection of blood into the SA space. All rats, were treated with buprenorphine (0.1 mg/kg), penicillin G (40 kilounits/kg) and hydrated with isotonic saline (5 ml, s.c.) and the allowed to recover.

**Figure 4:**
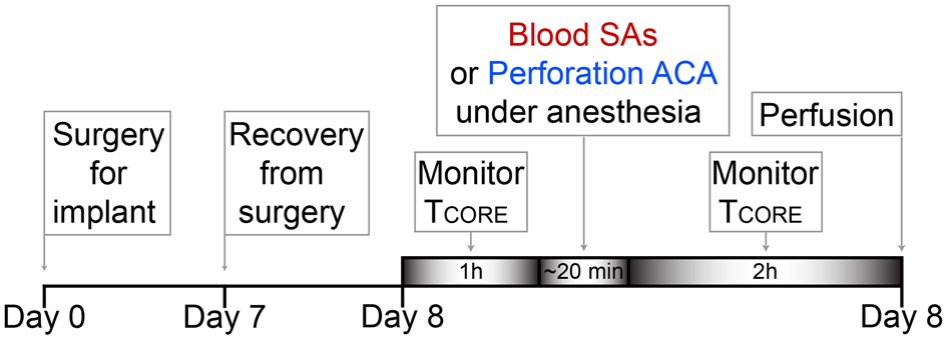
Schedule of experimental procedures. Timeline of the experimental protocol for (1) injection of blood into the subarachnoid space (SAs) and (2) perforation of the anterior cerebral artery (ACA). Rats underwent surgery for implantation of temperature recording devices on Day 0. On day 8, following 7 days of recovery from surgery, one hour of baseline core temperatures were recorded at 15 min intervals, followed by the procedure for blood injection into the SAs or perforation of the ACA. Core temperature was then monitored for 2 h, before perfusion, to assess the occurrence of NF.

On the day of the experiment, baseline T_CORE_ was monitored for 1 h (15 min intervals) before the injection procedure (Fig. 4), which was performed under anesthesia with 3% isoflurane in room air. With the rat supine, a rapid cannulation (no more than 10 min) of the femoral artery was performed to allow blood withdrawal (0.5 ml) for autologous injection of blood in the SA space. The rat was then placed in the stereotaxic frame with the incisor bar positioned − 4 mm below interaural zero. Blood was collected from the femoral artery, into a heparinized syringe, and then connected to a Whitacre needle for the SA injection procedure.

The cap of the Eppendorf, previously implanted on the skull, was then removed to gain access to the burr hole and the Whitacre needle was stereotactically inserted and lowered into the prechiasmatic cistern to perform blood injection into the SA space (see Cetas et al., 2009 for details). Control rats received the same surgical procedure and the same amount of blood withdrawal but they received a vehicle injection (aCSF) instead of blood into the SA space.

At the end of the injection, the femoral artery catheter was removed, the artery clamped with suture, and the skin incision sutured. Lidocaine was applied to the incision to reduce pain. Each rat recovered from anesthesia in a temperature-controlled and sound-attenuated room. T_CORE_ was then monitored for 2 h (15 min intervals) after the treatment (Fig. 4). Subsequently, rats were anesthetized with sodium phenobarbital, perfused with PBS (250 ml) and formaldehyde (250 ml, 4% in PBS. The brains were removed and post-fixed for 2 h in formaldehyde (4% in PBS).

### Surgical procedure and experimental protocol for perforation model

We used a perforation model to determine if the NF obtained with direct injection of blood into the SA space was comparable to that elicited by a model of the more natural SAH insult, the rupture of an aneurism. Due to technical limitations linked with the recording of BAT SNA, we couldn’t use the perforation model in all the experiments designed for this study. Seven days prior to the experiment, rats were anesthetized with 2% isoflurane in 100% O_2_ equipped with a temperature recording device (IPTT-300; Bio Medic Data Systems) to monitor temperature during the experimental procedures (Fig. 4). The temperature probe was implanted in the interscapular area, in close apposition to the dorsal muscle layer and beneath iBAT, in order to monitor temperature during the experimental procedures.

On the day of the experiment (Fig. 4), baseline T_CORE_ was monitored for 1 h (15 min interval) in rats maintained in a temperature-controlled and sound-attenuated room. Following the baseline recording, rats were anesthetized with 3% isoflurane in room air and placed supine on the stereotaxic frame to maintain the head in a fixed position during the procedure for the cannulation of the carotid artery which provided access to the anterior cerebral artery (ACA) for the arterial perforation procedure. A water perfused thermal blanket was used to maintain normothermia during this brief procedure (~ 20 min).

A skin and subcutaneous incision was performed in the right mediolateral side of the ventral surface of the neck, to gain access to the common carotid artery. The common, internal and external carotid arteries, were carefully dissected from the surrounding tissue while the pterygopalatine and thyroid arteries were cauterized to exclude blood flow and reduce the occurrence of hemorrhage during the procedures for cannulation of the internal carotid artery. Loose ties, made with a suture filament, were used to surround the common and internal carotid arteries to transiently block blood flow, while the external carotid artery was tied off completely and then severed. The open side of the external carotid artery was then used to gain access for a microfilament which was pushed through the internal carotid artery and the ACA until a resistance was felt. Perforation was then performed, by pushing the filament through the wall of the ACA. Following the perforation, the ties on the common and internal carotid artery were removed to reestablish the blood flow to the brain and induce a SAH. Confirmation of hemorrhage was assessed by sudden changes in HR, transient loss of respiration (data not reported) and then postmortem identification of blood in the SA space. Sham rat received the identical surgery, the filament was inserted to the internal carotid artery bifurcation with the ACA, but then retracted without causing an arterial perforation. Following the perforation procedure, the neck incision was sutured and treated with lidocaine to reduce pain.

Rats recovered from anesthesia in a temperature-controlled and sound-attenuated room. Following the treatment, T_CORE_ was monitored for 2 h (15 min interval) and then rats were anesthetized with sodium phenobarbital and perfused with PBS (250 ml) followed by formaldehyde (250 ml, 4% in PBS). Brain were removed and post-fixed for 2 h in formaldehyde (4% in PBS).

### Surgical procedure for BAT Synaptic Nerve Activity (SNA) recording

Rats were anesthetized initially with 3% isoflurane in 100% O_2_ and transitioned to urethane (0.8 g/kg) and chloralose (80 mg/kg) following cannulation of a femoral artery and vein. Heart rate (HR) was derived from the femoral arterial pressure (AP) signal. Animals were positioned prone in a stereotaxic frame with the incisor bar − 4 mm below interaural zero and a spinal clamp installed on the T10 vertebra to maintain the spine in a rigid and elevated position that provides (a) a constant and horizontal positioning of the caudal brainstem, (b) an optimal oil pool within which to record BAT SNA, and (c) a reduced potential for respiratory-related artifacts in the BAT SNA recording. The medial portions of the parietal and occipital bones were partially removed and the dura was dissected to allow the stereotaxic (AP − 1.2 mm; LL 1.5 mm; DV − 4 mm) insertion of an intracerebroventricular (ICV) cannula for ICV injection of drugs. A second burr hole, was drilled at the midline between the two frontal bones (7.5 mm anterior to Bregma) to allow the penetration of a Whitacre needle into the SA space at the base of the brain (as described in Cetas et al., 2009).

Rats were paralyzed with D-tubocurarine (0.3 mg initial dose, 0.1 mg/h supplements) and artificially ventilated via a tracheal cannula with 100% O_2_ (60–70 cycles/min, tidal volume 3–3.5 ml). Small adjustments in minute ventilation were made to maintain basal mixed-expired CO_2_ levels between 3.0% and 4.5%. Thermocouples (Physitemp Instruments with Sable Systems International meter) were placed on the shaved abdominal skin to measure skin temperature (T_SKIN_), 6 cm into the rectum to measure T_CORE_, and into the medial aspect of the left interscapular BAT (iBAT) pad to measure T_BAT_. A water perfused blanket surrounding the torso of the rat was used to adjust T_CORE_ and T_SKIN_ to the appropriate pretreatment temperatures required for the experiment. Prior to eliciting NF with injections into the SA space, T_SKIN_ and T_CORE_ were stabilized at ~ 37 °C, resulting in low control levels of BAT SNA. During the experimental manipulations (e.g., SA space injections), BAT SNA and T_BAT_ levels were allowed to fluctuate in response to the treatment.

Postganglionic BAT SNA was recorded from the central cut end of a small nerve bundle dissected from the ventral surface of the right iBAT pad after dividing the fat pad along the midline and reflecting it laterally. Nerve activity was recorded with bipolar hook electrodes, filtered (1–300 Hz), and amplified (20,000×; Cyberamp 380, Axon Instruments).

### Blood fractionation protocol

Rats were anesthetized with 3% isoflurane. The femoral artery was cannulated for acquisition of arterial blood pressure and blood. Blood (0.5 ml) was slowly withdrawn, to reduce platelet activation, and collected in a previously heparinized (50 µl heparin, 200 units/ml) 2 ml tube.

Collected blood was placed in a centrifuge (Sorval microfuge rotor 7500 3328) and spun at 300×*g* for 10 min to separate the four mains component of the blood: Plasma, platelets, white blood cells (WBC) and erythrocytes (RBC).

The supernatant containing plasma and platelets was carefully removed and collected in a vial for a second spin at 300×*g* for 10 min to ensure the complete remove of RBC from this blood fraction. Following the recovered plasma was stored at 4 °C before the use as it is for injection in the SA space.

WBC collected at the interface between plasma and RBC, were carefully removed by aspiration. The remaining RBC collected at the bottom were washed 3 times by resuspension in 1.5 ml of isotonic PBS and spun at 300×*g* for 10 min. Supernatant was recovered and disposed after each wash. To restore the initial hematocrit value of the blood, the pellet fraction containing RBC was re-suspended with isotonic PBS, to the 0.5 ml mark of the vials corresponding to the amount of initial blood withdrawn. The solution of re-suspended RBC was stored at 4 °C until needed for injection in the SA space. Tint plasma or tint washing medium was considered a sign of significant RBC rupture and release of hemoglobin. In these cases, the fractionated products were not used for experiments and a second blood sample was obtained to repeat the fractionation protocol.

### Drug injection procedure

For ICV injection of drugs, a metal cannula (PlasticsOne) connected to a 100 µl syringe (Hamilton), was lowered stereotactically into the left lateral ventricle and 5 µl of drug solution was injected over 2 min. After physiological recordings, rats were perfused transcardially with 0.9% isotonic saline, followed by 4% paraformaldehyde in phosphate-buffered saline (PBS). The brains were removed to determine the extent of the diffusion of the blood in the SA space.

### Drugs

N6-cyclohexyladenosine (CHA, Sigma Aldrich) was dissolved in isotonic saline to a concentration of 1 mM. Indomethacin (Sigma Aldrich) was dissolved in isotonic saline to a concentration of 2 mg/ml.

### Data acquisition

BAT SNA (1–300 Hz, 1 kHz), T_BAT_ (5 Hz), T_CORE_ (5 Hz), T_SKIN_ (5 Hz), T_PAW_ (5 Hz), expired CO_2_, AP (200 Hz), EKG (10–300 Hz, 1 kHz) and stimulus trigger pulse signals were filtered and digitized (Cyberamp, Axon Instruments; Micro 1401 MKII; Cambridge Electronic Design) and recorded onto a computer hard drive for subsequent analysis (Spike 2, CED). For the BAT SNA, continuous measures (4 s bins) of signal amplitudes were calculated as the root mean square (rms) value of the BAT SNA (square root of the total power in the 0.1–20 Hz band), from the autospectra of sequential 4-s segments for each signal.

### Data and statistical analysis

For analysis of physiological variables, the data were averaged into 30 s bins, and group data were reported as mean ± standard error of the mean (SEM). To account for slight differences in nerve recording characteristic among experiments, raw BAT SNA values in individual experiments were normalized to the minimum BAT SNA [i.e., baseline (BL)] recorded under warm conditions (T_CORE_ > 36 °C) and expressed as a percentage of this baseline value (% BL).

All statistics were performed using using GraphPad Prism (Version 6.00, www.graphpad.com). The statistical comparisons were performed using either Student t-test for which t value are reported, or repeated measure two-way ANOVA, followed by bonferroni post-hoc comparison used for individual comparison between a single control value at baseline vs. a single value at the peak of the post-treatment response. T and p values are reported for each comparison. Statistical results with p < 0.05 were considered significant.

## Data Availability

The data that support the findings of this study are available from the corresponding author upon reasonable request.
